# Transcriptomic analysis reveals the contribution of *QMrl-7B* to wheat root growth and development

**DOI:** 10.3389/fpls.2022.1062575

**Published:** 2022-11-14

**Authors:** Jiajia Liu, Liya Zhi, Na Zhang, Wei Zhang, Deyuan Meng, Aamana Batool, Xiaoli Ren, Jun Ji, Yanxiao Niu, Ruiqi Li, Junming Li, Liqiang Song

**Affiliations:** ^1^ Center for Agricultural Resources Research, Institute of Genetics and Developmental Biology, Chinese Academy of Sciences, Shijiazhuang, Hebei, China; ^2^ The College of Life Science, University of Chinese Academy of Sciences, Beijing, China; ^3^ Ministry of Education Key Laboratory of Molecular and Cellular Biology, Hebei Collaboration Innovation Center for Cell Signaling, Hebei Key Laboratory of Molecular and Cellular Biology, College of Life Sciences, Hebei Normal University, Shijiazhuang, China; ^4^ State Key Laboratory of North China Crop Improvement and Regulation, College of Agronomy, Hebei Agricultural University, Baoding, Hebei, China

**Keywords:** *Triticum aestivum*, root traits, RNA sequencing, differentially expressed gene, near-isogenic lines

## Abstract

Roots are the major organs for water and nutrient acquisition and substantially affect plant growth, development and reproduction. Improvements to root system architecture are highly important for the increased yield potential of bread wheat. *QMrl-7B*, a major stable quantitative trait locus (QTL) that controls maximum root length (MRL), essentially contributes to an improved root system in wheat. To further analyze the biological functions of *QMrl-7B* in root development, two sets of *Triticum aestivum* near-isogenic lines (NILs), one with superior *QMrl-7B* alleles from cultivar Kenong 9204 (KN9204) named NIL^KN9204^ and another with inferior *QMrl-7B* alleles from cultivar Jing 411 (J411) named NIL^J411^, were subjected to transcriptomic analysis. Among all the mapped genes analyzed, 4871 genes were identified as being differentially expressed between the pairwise NILs under different nitrogen (N) conditions, with 3543 genes expressed under normal-nitrogen (NN) condition and 2689 genes expressed under low-nitrogen (LN) condition. These genes encode proteins that mainly include $NO_{3}^{-}$ transporters, phytohormone signaling components and transcription factors (TFs), indicating the presence of a complex regulatory network involved in root determination. In addition, among the 13524 LN-induced differentially expressed genes (DEGs) detected in this study, 4308 and 2463 were specifically expressed in the NIL^KN9204^ and NIL^J411^, respectively. These DEGs reflect different responses of the two sets of NILs to varying N supplies, which likely involve LN-induced root growth. These results explain the better-developed root system and increased root vitality conferred by the superior alleles of *QMrl-7B* and provide a deeper understanding of the genetic underpinnings of root traits, pointing to a valuable locus suitable for future breeding efforts for sustainable agriculture.

## Introduction

Crops produce a large proportion of food and nutrients for humans in modern society. During the past 50 years, the application of chemical fertilizers and the Green Revolution have substantially increased crop production, maintaining food security for the increasingly growing population (Godfray et al., 2010). However, a continuous increase in crop output cannot sustainably be achieved by excessive amounts of fertilizer because of the limited nutrient use efficiency (NUE) of crop plants. Importantly, a large amount of wasted resources and environmental pollution resulting from excessive fertilizer applications cannot be ignored (Springmann et al., 2018). For these reasons, crop cultivars that have relatively high NUE are environmentally friendly and helpful to conserve resources for sustainable agriculture. Roots, the major organs for plant anchorage, nutrient and water absorption, and interactions with symbiotic organisms, have attracted increased amounts of attention in recent studies given the discovery of their correlations with nutrient use and crop yield (Gewin, 2010). In general, manipulation of root architecture traits, including root number, root length, root surface area (RA) and root volume (RV), especially the spatial configuration of the root system in the soil, is an essential approach to increase crop yields, which may represent an important component of a new green revolution (Lynch, 2007; Meister et al., 2014). Unfortunately, root traits have been chronically overlooked in the traditional breeding due to their lack of aboveground visibility (Maqbool et al., 2022).

Bread wheat (*Triticum aestivum* L.) is one of the most important crop species worldwide, providing ~20% of calories for human needs (Appels et al., 2018). Efforts have been made to explore the determinants of root traits that contribute to increase yield potential in wheat. In addition to traditional labor-intensive and time-consuming methods for root trait analysis (Kücke et al., 1995), artificial medium systems, such as hydroponic culture, have been used in many studies and revealed a series of quantitative trait loci (QTLs) that drive genetic variation in root traits (An et al., 2006; Cao et al., 2014; Kabir et al., 2015; Maccaferri et al., 2016; Merchuk-Ovnat et al., 2017; Fan et al., 2018; Zheng et al., 2019). However, it remains largely unclear of the mechanisms of these loci control root traits due to the complexity of the wheat genome. To date, a few genes regulating root morphology have been analyzed through reverse genetics studies in wheat. For example, the expressions of *TaRSL2* (Han et al., 2017a) and *TaLBD16* (Wang et al., 2018a) were shown to be positively correlated with root hair length and number of lateral roots, respectively. Heterogenous expressions of *TaMPK4* (Hao et al., 2015) and *TaMOR* (Li et al., 2016) demonstrated their positive effects on root development. Overexpression of the transcription factor (TF)-encoding genes *TaNAC2-5A* (He et al., 2015), *TaNFYA-B1* (Qu et al., 2015), *TaNAC69-1* (Chen et al., 2016) and *TaWRKY51* (Hu et al., 2018) resulted in enhanced root growth, while *TabZIP60* (Yang et al., 2019) had the opposite effects, implying a complex genetic basis of the formation of root traits. In addition, circular RNAs have been shown to participate in root length regulation (Xu et al., 2019).

At present, a holistic view of the root growth regulatory network and how it affects root traits and yield potential is strongly needed for molecular breeding. RNA sequencing (RNA-seq) makes it possible to explore the consistency between root morphology and its underlying mechanisms at the whole-plant level through DEG analysis. For example, the transcriptomic analysis revealed how the root growth of salt-tolerant bermudagrass was maintained under salinity stress; the salt tolerance was attributed to appropriate expression levels of genes involved in reactive oxygen species (ROS) homeostasis and cell wall loosening, which were regulated in part by a number of TFs linked to stress responses and growth regulation (Hu et al., 2015). DEGs detected in maize cultured under different nitrogen (N) levels highlighted the network of root growth in response to N deficiency, where the *LBD* TF-encoding gene participated in the balance between nutrient metabolism and the stress response (He et al., 2016). RNA-seq data suggested crosstalk occurs among the roots (in terms of growth promotion), gibberellin (GA) biosynthesis, auxin signaling, cell division and cell wall modification in uniconazole-treated soybean (Han et al., 2017b). These findings enriched our understanding of the molecular mechanisms behind the interplay of root traits and other agronomic characteristics, helping to generate strategies for crops with improved root systems, increased NUE and improved environmental adaptation.

Previous studies have shown that *Triticum aestivum* KN9204has a larger root system than does cultivar J411 (Wang et al., 2011). Using a RIL population derived from the cross of KN9204 and J411 (KJ-RIL), researchers identified *QMrl-7B* as a major stable QTL that controls maximum root length (MRL) (Fan et al., 2018), and its contribution to large root systems was dissected (Liu et al., 2021), prompting the question of how *QMrl-7B* regulates root architecture and N uptake. In the present study, transcriptomic analysis of the pairwise *QMrl-7B* NILs was performed to further analyze the function of *QMrl-7B* in root development. The results will help reveal the pathways controlling root development that are affected by *QMrl-7B* in wheat, providing insights into the genetic and physiological basis underlying desirable root traits for agriculture.

## Materials and methods

### Plant materials and root phenotyping

KN9204 is a representative cultivar released in 2003 with a prolific root system, and J411 is a major cultivar that has been planted in the Northern China Plain since 1990s (Wang et al., 2011). Two sets of *QMrl-7B* NILs, one with superior alleles from KN9204 (referred to as the NIL^KN9204^) and another with inferior alleles from J411 (referred to as the NIL^J411^) (Liu et al., 2021), were used in this study for transcriptomic analysis.

The root morphological traits of the pairwise *QMrl-7B* NILs were characterized in the experiments involving different N hydroponic cultures, with three replications. Seeds were imbibed in distilled water at 23°C for 24 h and then transferred to plastic pots containing moist vermiculite and nutrient-enriched soil (1:1, v/v) for ten days. Uniform seedlings (at the one- and half-leaf stages) were then chosen, after which their residual endosperm was removed; they were subsequently cultured in both normal-nitrogen (NN) and low-nitrogen (LN) nutrient solutions as described by Fan et al. (2018). On the fourteenth (four-leaf stage) and twenty-first (five-leaf stage) days in different N hydroponic cultures, five uniform seedlings of each NIL were evaluated for their average MRL (cm), total root length (TRL, cm), RA (cm^2^) and RV (cm^3^). MRL was measured using a ruler, and the remaining traits (TRL, RA and RV) were quantified by using a ScanMaker i800 Plus Scanner (600 DPI) and analyzed by LA-S software (Hangzhou Wanshen Detection Technology Co., Ltd., Hangzhou, China; https://www.wseen.com).

Moreover, the roots of the four-leaf-stage seedlings of the pairwise NILs were sampled and immediately frozen in liquid N for subsequent RNA extraction. The root samples were divided into four treatments: the NIL^KN9204^ under NN condition (NN_NIL^KN9204^), the NIL^J411^ under NN condition (NN_NIL^J411^), the NIL^KN9204^ under LN condition (LN_NIL^KN9204^) and the NIL^J411^ under LN condition (LN_NIL^J411^). Each sample pool comprised five individual seedlings, and all experiments were replicated three times.

### Transcriptome analysis

#### RNA extraction and sequencing

Total RNA was extracted from the root samples using TRIzol Reagent (Plant RNA Purification Reagent for plant tissue, Invitrogen, USA) according to the manufacturer’s instructions, and genomic DNA was removed using DNase I (TaKaRa, Beijing, China). The quantity, quality, and integrity of the extracted RNA were determined using an ND-2000 spectrophotometer (Thermo Fisher Scientific, MA, USA) and an Agilent 2100 Bioanalyzer (Agilent Technologies, Santa Clara, CA, USA). Only high-quality RNA samples (OD260/280 = 1.8~2.2, OD260/230 ≥ 2.0, RNA integrity number (RIN) ≥ 6.5, 28S:18S ≥ 1.0, RNA content > 1 μg) were used to construct a sequencing library. Messenger RNA (mRNA) was isolated from the total RNA using magnetic beads with oligo (dT) primers and then cut into short fragments in a fragmentation buffer. Complementary DNA (cDNA) was subsequently synthesized using a SuperScript Double-Stranded cDNA Synthesis Kit (Invitrogen, CA, USA), with random hexamer primers (Illumina). The synthesized cDNA was then subjected to end repair, phosphorylation and polyadenylation according to Illumina’s library construction protocol. The libraries were size selected for cDNA target fragments of 300 bp on 2% low-range ultra-agarose followed by PCR amplification using Phusion DNA polymerase (NEB); fifteen PCR cycles were performed. After quantification by a TBS-380 fluorometer, the paired-end RNA-seq library was sequenced with an Illumina HiSeq Xten/NovaSeq 6000 sequencer (2 × 150 bp read length). The library was then sequenced using the Illumina HiSeq™ platform (Shanghai Majorbio Bio-pharm Technology Co., Ltd., Shanghai, China).

#### Analysis of DEGs

The data were analyzed on the free online platform of the Majorbio Cloud Platform (https://www.majorbio.com). To obtain high-quality reads, reads containing adapter sequences and reads of low quality were removed from the raw data to obtain clean reads in FASTQ format. Q20 and Q30 values and GC contents were determined according to conventional methods. Afterwards, the remaining clean reads were mapped to the reference genome of Chinese Spring (CS) (http://plants.ensembl.org/Triticum_aestivum/Info/Index/) by HISAT2 software (http://ccb.jhu.edu/software/hisat2/index.shtml). Unigene expression levels were estimated according to their transcripts per million reads mapped (TPM) values. DEGs between the NIL^KN9204^ and the NIL^J411^ under both NN and LN conditions were then analyzed using the DESeq2 package (Love et al., 2014), and the expression results are presented in terms of the log_2_fold change (FC). |log_2_FC| ≥ 1 and *p*-adjusted< 0.05 were used as thresholds to judge the significance of gene expression differences. DEGs were also evaluated in response to LN stress for each NIL.

The DEGs of each one-to-one comparison were analyzed by Gene Ontology (GO) and Kyoto Encyclopedia of Genes and Genomes (KEGG) pathway enrichment analysis. GO functional enrichment and KEGG pathway analysis were carried out by Goatools (https://github.com/tanghaibao/Goatools) and KOBAS (http://kobas.cbi.pku.edu.cn/home.do), where GO terms and biological pathways with a *p*-adjusted< 0.05 were considered significantly enriched. The encoded products of the DEGs were subsequently annotated by the NCBI non-redundant protein (NR) database.

### Quantitative real-time polymerase chain reaction validation

To confirm the reliability of the DEGs obtained from RNA-seq, eight DEGs on chromosome 7B were selected for qRT-PCR analysis. Gene-specific primers were designed using Primer Premier 5.0 software (Premier Biosoft International); the sequences are shown in **Table S1**. qRT-PCR was carried out in total volumes of 20 μl using a SYBR PCR kit (TaKaRa, Beijing, China) on an ABI 7500 real-time PCR system (Applied Biosystems, MA, USA) according to the manufacturers’ instructions. Each sample was analyzed as three technical replicates, and the relative expression value of the DEGs was determined using the comparative cycle threshold (Ct) method after normalization to the expression of the GAPDH control.

## Results

### Root traits of the pairwise NILs

**Figure 1:**
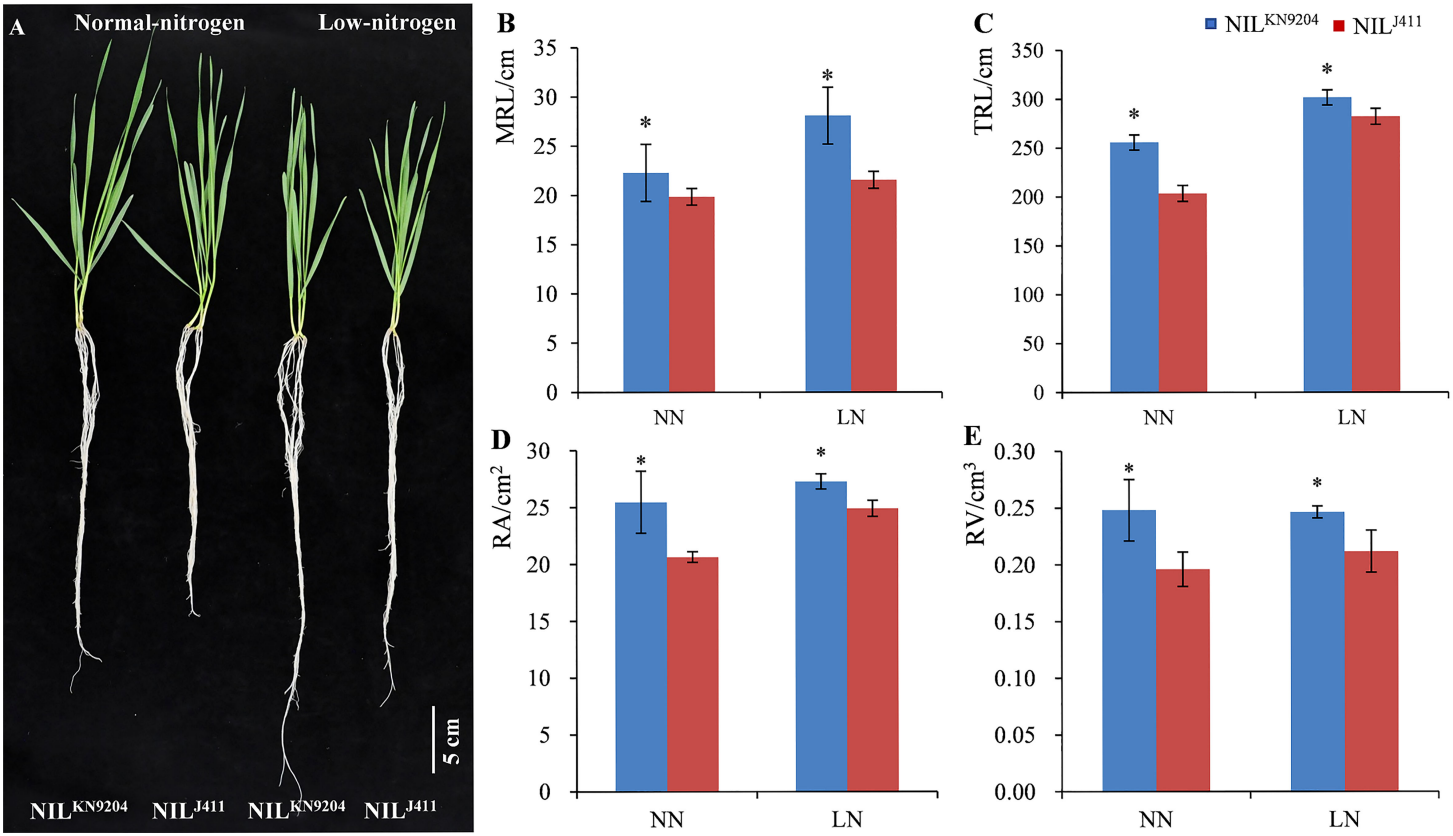
Phenotypes of four-leaf-stage seedlings **(A)** and the corresponding root traits **(B-E)** of the pairwise NILs cultured in nutrient solutions with different N contents for fourteen days NN and LN indicate normal- and low-nitrogen conditions, respectively. MRL, TRL, RA and RV indicate the maximum root length, total root length, root surface area and root volume, respectively. “*” indicates a significant difference at *p*< 0.05.

The root traits of the seedlings in hydroponic cultures with different N contents were evaluated. On the fourteenth day after the seedlings were transferred to the nutrient solutions, the average MRL, TRL, RA and RV of the four-leaf-stage NIL^KN9204^ seedlings were 22.28 cm, 255.77 cm, 25.47 cm^2^ and 0.25 cm^3^, respectively, under NN condition, which were significantly greater than those of the NIL^J411^ (19.85 cm, 203.47 cm, 20.64 cm^2^ and 0.20 cm^3^, respectively) under comparable conditions. Accordingly, the average MRL, TRL, RA and RV of the NIL^KN9204^ *vs* NIL^J411^ at the seedling stage were 28.07 *vs* 21.54 cm, 301.75 *vs* 282.12 cm, 27.28 *vs* 24.90 cm^2^ and 0.25 *vs* 0.21 cm^3^, respectively, under LN condition, indicating that the NIL^KN9204^ was superior to the NIL^411^ by 30.32%, 6.96%, 9.56% and 19.05%, respectively (*p*< 0.05). Notably, the MRL of the NIL^KN9204^ seedlings under LN condition was 25.99% greater than that under NN conditions, while only an 8.51% difference was recorded for the NIL^411^ under the hydroponic cultures with different N contents, indicating that LN more strongly induced the growth of primary roots for the NIL^KN9204^ than for the NIL^411^ (**Figure 1**). Similar change trends of MRL, TRL, RA and RV differences in the five-leaf-stage seedlings were also observed between the two sets of NILs at twenty-one days after the 10-day-old seedlings were transferred to the nutrient solutions (**Figure S1**). Taken together, the above results showed that, in comparison to the NIL^411^, the NIL^KN9204^ had superior root architecture characteristics during the seedling stage, especially under LN condition.

### Overview of transcriptome sequencing results

To explore the genetic and physiological basis of the superior root traits of the NIL^KN9204^, roots of four-leaf-stage seedlings of the pairwise NILs cultured under both NN conditions and LN conditions were sampled for transcriptomic analysis. RNA-seq on the Illumina HiSeq™ platform (http://www.majorbio.com) generated 70576620~90524786 clean reads per sample, with a sequencing error of less than 0.3%. The average Q20, Q30 and GC content percentages were 97.06%, 92.27% and 53.98%, respectively. These findings attest to the fine quality of the sequencing results. Mapping of the reads with the Chinese Spring (CS) reference genome revealed an alignment in a range of 81.48~90.35%, among which 76.14~84.54% of the genes were uniquely mapped and 5.34~6.28% were multiply mapped (**Table S2**). The correlation coefficient of repeats ranged from 0.69~0.98 (**Figure S2**, **Table S3**). These data were deemed suitable for subsequent analysis (**Table S4**), in which the DEGs whose expression was upregulated (log_2_FC ≥ 1) and downregulated (log_2_FC ≤ −1) in each one-to-one comparison were statistically (*p*-adjusted< 0.05) analyzed.

### Identification of DEGs between the pairwise NILs

**Figure 2:**
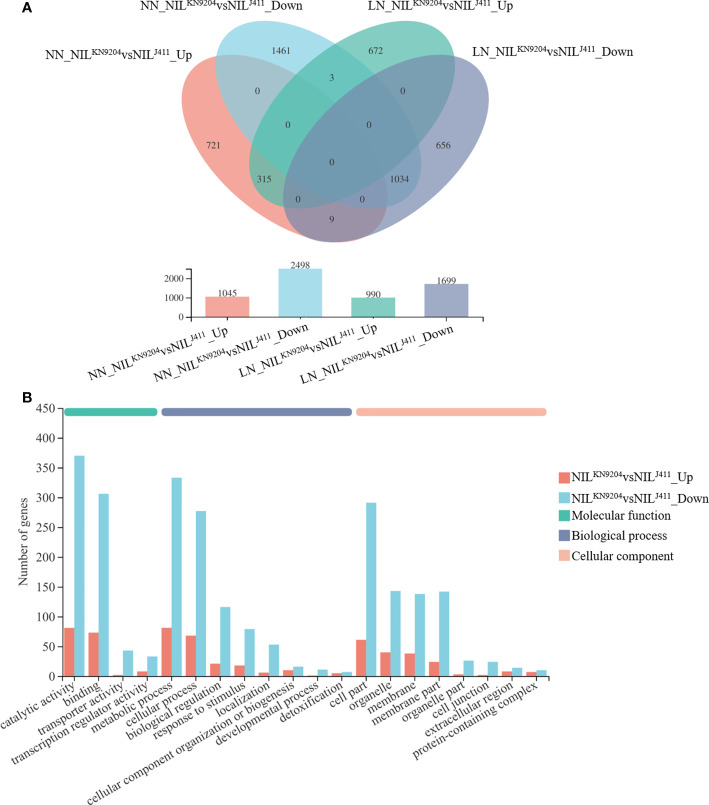
Identification of DEGs between the pairwise NILs NN and LN indicate normal- and low-nitrogen conditions, respectively. The expression levels between the NIL^KN9204^ and the NIL^J411^ were compared under each condition, with the former as a control. NIL^J411^ *vs*NIL^KN9204^_Up and NIL^J411^ *vs*NIL^KN9204^_Down indicate DEGs whose expression was upregulated and downregulated in the NIL^J411^ compared to the NIL^KN9204^ under the same culture conditions, respectively. **(A)** Venn diagram analysis of DEGs in each one-to-one comparison between the pairwise NILs under both NN and LN conditions. **(B)** GO annotation analysis of DEGs between the pairwise NILs that were simultaneously expressed under both NN and LN conditions with the first 20 terms as indicated.

The transcriptomic changes in the roots between the pairwise *QMrl-7B* NILs were compared under both NN and LN conditions. Under NN condition, a total of 3543 genes were differentially expressed between the two sets of NILs, of which the expression of 1045 genes was upregulated and that of 2498 was downregulated in the NIL^KN9204^ (**Figure 2A**; **Table S5**). These genes were enriched in 320 subcategories according to GO analysis, of which 200, fourteen and 106 belonged to biological processes, cellular components and molecular functions, respectively, and the three most enriched biological processes were “biological process (GO:0008150)”, “metabolic process (GO:0008152)” and “phosphate-containing compound metabolic process (GO:0006796)” (**Table S6**). “Phenylpropanoid biosynthesis (map00940)” was the most enriched pathway according to KEGG analysis (**Table S7**). Under the LN condition, 2689 genes were differentially expressed between the pairwise NILs, including 990 genes whose expressions were upregulated and 1699 genes whose expressions were downregulated in the NIL^KN9204^ (**Figure 2A**; **Table S8**). These DEGs were categorized into biological process (141), cellular component (12) and molecular function (77) categories by GO analysis. The three most enriched biological processes were “biological process (GO:0008150)”, “metabolic process (GO:0008152)” and “phosphate-containing compound metabolic process (GO:0006796)” (**Table S9**). Similarly, “phenylpropanoid biosynthesis (map00940)” was the most enriched pathway according to the KEGG analysis (**Table S10**).

Notably, there were 315 DEGs whose expressions increased and 1034 DEGs whose expressions decreased in the NIL^KN9204^ compared to the NIL^J411^ simultaneously under both NN and LN conditions (**Figure 2A**). These genes might participate in translation, transcriptional regulation and cycle control according to GO and KEGG enrichment analysis (**Figure 2B**; **Tables S11-S13**), reflecting the permanent effects of the *QMrl-7B* locus that were not affected by N levels. Under NN condition, nine genes were expressed at higher levels in the NIL^KN9204^ than in the NIL^J411^; however, these genes were expressed at a higher level in the NIL^J411^ under LN condition (**Figure 2A**; **Table 1**). In addition, three genes exhibited the opposite expression trend, implying their functional difference in response to N stress (**Figure 2A**; **Table 1**).

**Table 1:** DEGs with opposite expression patterns between the pairwise NILs under NN and LN conditions.

Gene_ID	log_2_FC (NN_NIL^KN9204^/NN_NIL^J411^)	*p*-adjusted	log_2_FC (LN_NIL^KN9204^/LN_NIL^J411^)	*p*-adjusted	NR_description
*TraesCS2A02G399900*	1.07	0.002	-1.97	7.77E-20	MATE efflux family protein 9
*TraesCS2B02G125500*	-1.37	0.0098	1.08	1.25E-06	class III peroxidase
*TraesCS2B02G626400*	-5.11	2.88E-34	1.11	2.48E-05	probable 1-deoxy-D-xylulose-5- phosphate synthase 2, chloroplastic
*TraesCS3B02G448000*	1.74	1.07E-18	-1.35	1.98E-06	unnamed protein product
*TraesCS3D02G094500*	-2.06	5.54E-07	1.68	0.001	MYB-related protein 305-like
*TraesCS3D02G225500*	1.16	0.0002	-1.42	4.08E-14	uncharacterized protein LOC109770081
*TraesCS3D02G408000*	1.21	2.62E-07	-1.68	4.76E-16	BTB/POZ and TAZ domain-containing protein 2-like
*TraesCS4A02G286600*	1.32	4.98E-05	-1.73	2.50E-10	uncharacterized protein LOC109755115
*TraesCS4B02G078800*	1.85	3.67E-15	-1.07	5.58E-06	hypothetical protein TRIUR3_28321
*TraesCS4D02G025500*	1.18	0.0003	-1.97	2.85E-08	uncharacterized protein LOC109755115
*TraesCS6B02G044300*	1.15	0.001	-1.47	4.78E-08	high-affinity nitrate transporter TaNRT2
*TraesCS6D02G035900*	1.01	0.016	-1.02	0.002	high-affinity nitrate transporter 2.1-like

NN and LN indicate normal- and low-nitrogen conditions, respectively. AA and BB indicate the NIL^KN9204^ and the NIL^J411^, respectively. The expression levels between the NIL^KN9204^ and the NIL^J411^ were compared under each condition, with the latter as a control. The expression ratios are presented as log_2_FC values. Genes with a log_2_FC ≤ -1 (p-adjusted< 0.05, coloured blue) represent the genes whose expression was downregulated in the NIL^KN9204^, and genes with log_2_FC ≥ 1 (p-adjusted< 0.05, coloured red) represent the genes whose expression was upregulated in the NIL^KN9204^.

### Identification of DEGs in response to N deficiency

**Figure 3:**
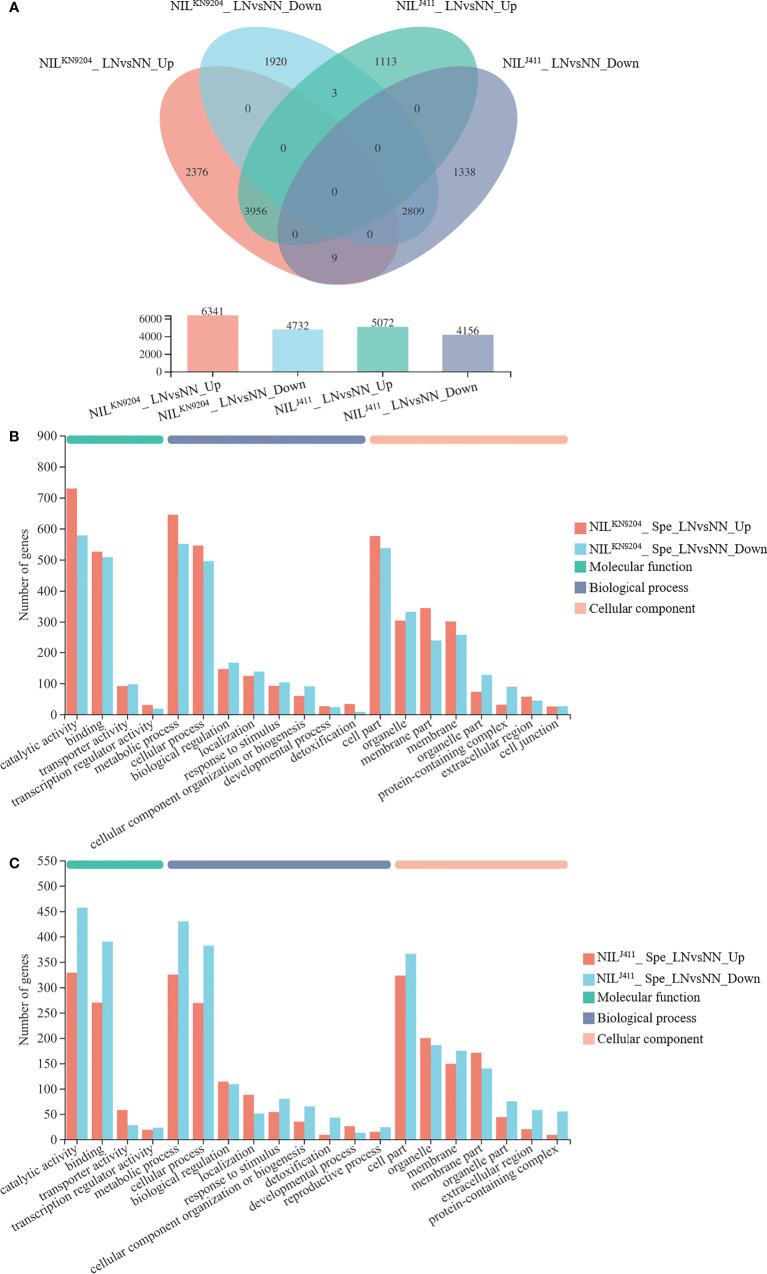
Identification of DEGs induced by LN NN and LN indicate normal- and low-nitrogen conditions, respectively. Spe indicates DEGs specifically expressed in the NIL^KN9204^ or the NIL^J411^. The expression levels between NN and LN conditions were compared for each NIL, with NIL^KN9204^ as a control. LN*vs*NN_Up and LN*vs*NN_Down indicate DEGs whose expression was upregulated and downregulated under LN compared to NN for the same NIL. **(A)** Venn diagram analysis of the DEGs in each one-to-one comparison between NN and LN conditions for both the NIL^KN9204^ and NIL^J411^. **(B, C)** GO annotation analysis of the genes whose expression differed specifically in the NIL^KN9204^ **(B)** and NIL^J411^ **(C)** with the first 20 terms as indicated.

The differences in DEGs in the root response to N deficiency between the NIL^KN9204^ and NIL^J411^ were analyzed. Compared with the NN condition, LN condition yielded 11073 DEGs (6341 whose expression increased and 4732 whose expression decreased) and 9228 DEGs (5072 whose expression increased and 4156 whose expression decreased) in the NIL^KN9204^and NIL^J411^, respectively (**Figure 3A**). A total of 2385 DEGs whose expressions increased and 1923 DEGs whose expressions decreased were detected only in the NIL^KN9204^ (**Figure 3A**; **Table S14**), whereas 1116 DEGs whose expressions increased and 1347 DEGs whose expressions decreased were detected specifically in the NIL^J411^ (**Figure 3A**; **Table S17**). Interestingly, there were more genes whose expressions were upregulated than genes whose expressions were downregulated in the NIL^KN9204^, and the opposite trend occurred in the NIL^J411^. GO analysis showed that these DEGs participate in multiple processes, and “phenylpropanoid biosynthesis (map00940)” was the most enriched process according to KEGG analysis (**Figures 3B, C**; **Tables S14-S19**). The expression of genes that differed specifically in the NIL^KN9204^ or the NIL^J411^ might give rise to a specific N response related to *QMrl-7B*, while a large number of common DEGs (3956 genes whose expression was upregulated and 2809 genes whose expression downregulated) in the two sets of NILs reflected the general response to N stress in wheat.

### Quantitative real-time PCR verification

**Figure 4:**
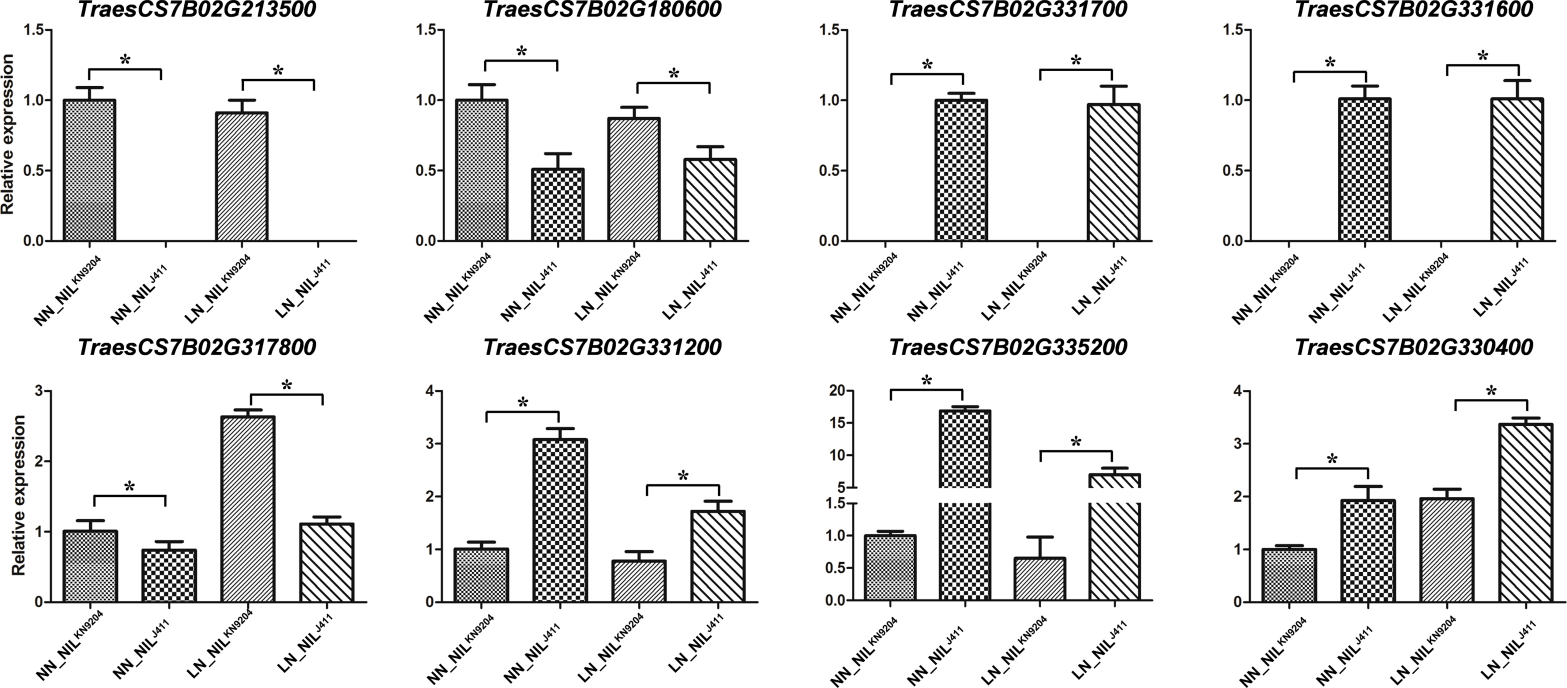
Validation of gene expression by qRT-PCR The relative mRNA expression of eight representative genes was checked by qRT-PCR. Each bar shows the mean ± standard error of three biological replicates. “*” indicates a significant difference at *p*< 0.05.

qRT**-**PCR analysis revealed that the expression levels of eight randomly selected DEGs on chromosome 7B were significantly different between the NIL^KN9204^ and the NIL^J411^ under NN conditions and LN conditions (**Figure 4**). These results nearly matched the expression profiles revealed by RNA-seq, confirming the results of the transcriptome sequencing of the pairwise NILs cultured under both NN and LN conditions.

## Discussion

Improving plant roots is an effective way to increase NUE and minimize fertilizer application for sustainable agricultural development (Lynch, 2007). A number of genes/QTLs have been reported to be related to root growth and development; as such, these genes/QTLs have potential application in crop genetic improvement (Zhuang et al., 2021; Chen et al., 2022; Li et al., 2022). However, the genetic network explaining how these genes interact to influence root architecture still needs to be elucidated to assess the beneficial agronomic traits from an overall perspective and to guide the use of beneficial genes or pyramid of allelic variation in future breeding efforts. Compared with J411, KN9204 had a larger root system (Wang et al., 2011), with which the stable QTL *QMrl-7B* was associated (Fan et al., 2018). Further studies involving *QMrl-7B* NILs showed that, as compare to the NIL^J411^s, the NIL^KN9204^s had superior root traits, N-related traits and yield traits in field trials (Liu et al., 2021). The superiority of root traits of the NIL^KN9204^ at the seedling stage was also consistently proven in the present study. When these hydroponically cultured pairwise NILs were used for transcriptomic analysis, it was found that the DEGs detected by RNA-seq encoded proteins of various groups, such as transporters, enzymes, TFs, and components involved in hormone signaling. The important DEGs that might be involved in root trait regulation are discussed here.

The genes with opposite expression patterns between the pairwise NILs under the NN and LN conditions were very likely to be involved in N-related root growth (**Tables 1**, **S5** and **S8**). For example, the expression level of the class III peroxidase gene (*TraesCS2B02G125500*) was higher in the NIL^J411^ under NN condition but was higher in the NIL^KN9204^ under LN condition. Class III peroxidase uses H_2_O_2_ as a substrate to produce OH^-^ radicals that enhance cell wall plastic extensibility and elongation by nonenzymatic cleavage of cell wall polysaccharides (Cosio and Dunand, 2009). Since root development is directly related to cell wall loosening and elongation, the upregulated expression of this gene by LN in the NIL^KN9204^ (**Table S14**) was proposed to participate in LN-promoted root growth by affecting these processes. Another most enriched process of the DEGs between the pairwise NILs was phenylpropanoid biosynthesis that might affect root enlargement (Muro-Villanueva et al., 2019; Liu et al., 2020). Interestingly, the expression levels of a series of phenylalanine ammonia-lyase and cinnamoyl-CoA reductase genes increased in the NIL^J411^ under both NN and LN conditions (**Tables S5** and **S8**), implying that the biosynthesis of cinnamate and lignin monomers increased, which cannot directly explain the reason for the smaller roots.

Nitrate is substantially absorbed and transported by high-affinity (NRT2s) and low-affinity (NPFs) nitrate transporters for assimilation and storage (Xu et al., 2012; Wang et al., 2018b). N stress tolerant sorghum genotypes exhibit higher expression levels of high-affinity NRTs than do the sensitive genotypes under N-limiting conditions (Gelli et al., 2014). We previously compared the expression profiles of 44 NRT2 and 217 NPF genes in the roots of KN9204 and J411 cultivated hydroponically with and without N supply and found that up to 79.2% and 43.7% of the root-expressed NRT2 and NPF genes, respectively, displayed differential expression between KN9204 and J411. Under NN conditions, more than two-thirds of the differentially expressed NRT2 and NPF genes exhibited higher expression in KN9204 than in J411, but the proportion of NRT2 and NPF genes with higher expression levels in KN9204 decreased under LN treatment, as the upregulated expression levels of most nitrate transporter genes, NRT2 family in particular, were higher in J411 than in KN9204 (Shi et al., 2022). Similarly, two high-affinity NRT genes (*TraesCS6B02G044300* and *TraesCS6D02G035900*) exhibited lower expression levels in the NIL^J411^ under NN condition, but higher expression levels in the NIL^J411^ than in the NIL^KN9204^ under LN condition (**Table 1**). In addition, the expressions of four high-affinity NRT genes (*TraesCS6A02G031000*, *TraesCS6A02G031100*, *TraesCS6B02G044100* and *TraesCS6B02G044500*) were induced specifically by LN in the NIL^J411^ but not in the NIL^KN9204^ (**Table 2**). Our present study showed that LN led to altere expression of a number of genes encoding both low-affinity and high-affinity NRTs (**Table S4**), in accordance with the viewpoint that different NRTs function at different N levels (Zhang and Forde, 1998).

**Table 2:** *NRT* genes specifically expressed in the NIL^KN9204^ or NIL^J411^ in response to LN.

Gene_ ID	log_2_FC (LN_NIL^KN9204^/NN_NIL^KN9204^)	*p*-adjusted	log_2_FC (LN_NIL^J411^/NN_NIL^J411^)	*p*-adjusted	NR_description
*TraesCS1A02G150200*	1.03	0.004	0.14	0.84	Peptide transporter PTR3-A
*TraesCS1A02G150400*	1.18	2.29E-07	0.64	0.08	protein NRT1/PTR FAMILY 5.2-like
*TraesCS1B02G168100*	1.60	1.11E-12	0.90	0.01	protein NRT1/PTR FAMILY 5.2-like
*TraesCS1B02G225000*	-1.39	2.12E-06	-0.14	0.81	protein NRT1/PTR FAMILY 6.3-like
*TraesCS1B02G267900*	1.29	0.01	1.15	0.18	protein NRT1/PTR FAMILY 3.1-like
*TraesCS1D02G201100*	1.13	2.34E-11	0.81	0.0004	uncharacterized protein LOC109744571
*TraesCS1D02G214300*	-1.47	7.24E-08	0.20	0.72	protein NRT1/PTR FAMILY 6.3-like
*TraesCS2A02G007500*	0.60	0.08	1.05	1.54E-09	protein NRT1/PTR FAMILY 8.5-like
*TraesCS2A02G074800*	-1.89	0.003	-0.58	0.45	high-affinity nitrate transporter 2.1-like
*TraesCS2A02G264500*	0.99	8.86E-05	1.06	0.0006	protein NRT1/PTR FAMILY 4.5-like
*TraesCS2B02G277600*	0.71	0.003	1.27	6.40E-09	protein NRT1/PTR FAMILY 4.5-like
*TraesCS2B02G626000*	1.02	1.30E-05	0.43	0.19	protein NRT1/PTR FAMILY 5.10-like
*TraesCS2B02G626700*	1.76	1.07E-08	0.97	0.01	protein NRT1/PTR FAMILY 5.10-like
*TraesCS2D02G413900*	1.50	0.02	1.74	0.08	protein NRT1/PTR FAMILY 8.2-like
*TraesCS2D02G583500*	0.99	0.01	1.30	0.0005	protein NRT1/PTR FAMILY 5.10-like
*TraesCS3A02G382100*	-2.55	6.06E-20	-0.94	0.34	protein NRT1/PTR FAMILY 5.1-like isoform X1
*TraesCS3A02G382200*	-0.12	0.90	1.57	0.003	unnamed protein product
*TraesCS3B02G095900*	0.20	0.62	1.12	0.02	unnamed protein product
*TraesCS3B02G414900*	-3.35	5.34E-07	0.20	0.90	unnamed protein product
*TraesCS3B02G415700*	0.81	0.12	1.01	0.02	predicted protein
*TraesCS3D02G056700*	0.85	0.25	1.43	0.048	protein NRT1/PTR FAMILY 8.2-like isoform X1
*TraesCS4A02G225400*	-1.07	6.53E-06	-0.97	0.007	protein NRT1/PTR FAMILY 4.3-like
*TraesCS4A02G283900*	1.20	6.27E-15	0.93	9.83E-06	protein NRT1/PTR FAMILY 2.11-like
*TraesCS4D02G026800*	1.01	1.57E-08	0.85	1.76E-05	protein NRT1/PTR FAMILY 2.11-like
*TraesCS4D02G087900*	-1.39	2.77E-07	-0.997	0.002	protein NRT1/PTR FAMILY 4.3-like
*TraesCS5B02G039100*	3.41	0.02	2.02	0.22	protein NRT1/PTR FAMILY 2.11-like
*TraesCS5B02G152000*	1.42	0.31	3.72	0.04	putative nitrate excretion transporter 3
*TraesCS5B02G245300*	2.48	8.76E-05	1.89	0.09	predicted protein
*TraesCS5B02G393100*	1.46	0.39	4.38	0.03	protein NRT1/PTR FAMILY 4.5-like
*TraesCS5B02G414000*	0.79	0.008	1.14	2.22E-05	protein NRT1/PTR FAMILY 6.4 isoform X2
*TraesCS5B02G498700*	-0.82	0.02	-1.09	0.01	protein NRT1/PTR FAMILY 5.6-like
*TraesCS5D02G419200*	0.89	0.03	1.91	0.0007	protein NRT1/PTR FAMILY 6.4 isoform X1
*TraesCS5D02G498700*	-1.03	0.009	-0.95	0.0004	protein NRT1/PTR FAMILY 5.6-like
*TraesCS6A02G030700*	-2.93	7.26E-18	-0.63	0.17	high-affinity nitrate transporter 2.1-like
*TraesCS6A02G030800*	-2.84	1.38E-27	-0.38	0.50	high-affinity nitrate transporter 2.1-like
*TraesCS6A02G030900*	-2.75	1.36E-29	-0.43	0.33	High-affinity nitrate transporter 2.1
*TraesCS6A02G031000*	0.20	0.78	1.63	0.0002	High-affinity nitrate transporter 2.1
*TraesCS6A02G031100*	0.01	0.98	1.45	3.00E-05	high affinity nitrate transporter
*TraesCS6A02G033100*	-6.88	4.09E-06	-2.71	1	high-affinity nitrate transporter 2.1-like
*TraesCS6A02G033200*	-6.79	8.35E-06	-3.36	1	High affinity nitrate transporter 2.4
*TraesCS6A02G267700*	0.30	0.62	-1.13	4.90E-05	protein NRT1/PTR FAMILY 8.3-like
*TraesCS6A02G369900*	4.75	0.01	0.22	0.93	protein NRT1/PTR FAMILY 8.3-like isoform X1
*TraesCS6B02G044100*	0.06	0.90	1.51	4.09E-05	high affinity nitrate transporter
*TraesCS6B02G044200*	-1.21	2.49E-05	0.42	0.39	high-affinity nitrate transporter 2.1-like
*TraesCS6B02G044400*	-1.37	6.30E-07	0.93	0.01	high-affinity nitrate transporter 2.1-like
*TraesCS6B02G044500*	0.17	0.72	3.46	2.47E-36	high affinity nitrate transporter
*TraesCS6B02G309200*	-1.09	0.10	-2.04	0.008	protein NRT1/PTR FAMILY 7.3-like
*TraesCS6D02G035900*	-2.25	6.07E-16	-0.22	0.69	high-affinity nitrate transporter 2.1-like
*TraesCS6D02G037800*	-3.88	0.02	-3.59	0.23	high-affinity nitrate transporter 2.1-like
*TraesCS6D02G132100*	1.45	0.008	1.18	0.09	protein NRT1/PTR FAMILY 8.3-like
*TraesCS7A02G121600*	1.87	4.14E-10	0.60	0.24	protein NRT1/PTR FAMILY 2.3-like
*TraesCS7A02G301700*	-1.26	5.33E-12	-0.96	3.48E-08	protein NRT1/PTR FAMILY 6.3-like
*TraesCS7B02G262200*	0.99	2.59E-05	1.16	2.16E-08	protein NRT1/PTR FAMILY 4.6-like
*TraesCS7B02G283400*	0.93	0.0001	1.31	0.0001	protein NRT1/PTR FAMILY 8.3-like
*TraesCS7D02G049300*	0.71	0.16	1.80	0.004	protein NRT1/PTR FAMILY 2.3-like
*TraesCS7D02G357300*	1.11	2.26E-06	0.83	0.0003	protein NRT1/PTR FAMILY 4.6-like

NN and LN indicate normal- and low-nitrogen conditions, respectively. AA and BB indicate the NIL^KN9204^ and the NIL^J411^, respectively. The expression levels between the NIL^KN9204^ and the NIL^J411^ were compared under each condition, with the latter as a control. The expression ratios are presented as log_2_FC values. Genes with a log_2_FC ≤ -1 (p-adjusted< 0.05, coloured blue) represent the genes whose expression was downregulated under LN conditions, and genes with log_2_FC ≥ 1 (p-adjusted< 0.05, coloured red) represent the genes whose expression was upregulated under LN conditions.

Dual-affinity nitrate transporter 1 (NRT1) not only functions in N acquisition but also is a $NO_{3}^{-}$ sensor; this action is independent of its uptake activity that controls root development (Ho et al., 2009). The cotransport of $NO_{3}^{-}$ , peptides and hormones through the NRT1 family of transporters implies the occurrence of crosstalk between $NO_{3}^{-}$ and other signaling pathways (Krouk et al., 2010; Tal et al., 2016). In our study, the expression levels of two and eleven putative NRT1 genes in the NIL^KN9204^ were higher and lower than those in the NIL^J411^ under both NN and LN conditions (**Table 3**), respectively, indicating that the differences in root traits may result mainly from NRT1 functional differentiation. Additionally, the higher expression level of the CBL-interacting protein kinase 23 (CIPK23) gene (*TraesCS2A02G251800*) in the NIL^J411^ compared to the NIL^KN9204^ was also independent of N status (**Tables S5** and **S8**); this phenomenon may affect root formation together with NRT1 by phosphorylating T101 to regulate its primary $NO_{3}^{-}$ response and high-affinity $NO_{3}^{-}$ transport (Ho et al., 2009). Taken together, these results implied that a more sensitive response of roots to N acquisition occurred in the NIL^J411^ compared with the NIL^KN9204^, which cannot be fully explained.

**Table 3:** Differentially expressed *NRT* genes between the pairwise NILs.

Gene_ ID	log_2_FC (NN_NIL^KN9204^/NN_NIL^J411^)	*p*-adjusted	log_2_FC (LN_NIL^KN9204^/LN_NIL^J411^)	*p*-adjusted	NR_description
*TraesCS2A02G309100*	-1.43	9.78E-12	-1.24	2.60E-12	protein NRT1/PTR FAMILY 4.3-like
*TraesCS2A02G572200*	1.13	0.04	1.20	0.003	protein NRT1/PTR FAMILY 5.10-like
*TraesCS5A02G485200*	-1.03	0.006	-1.12	0.01	protein NRT1/PTR FAMILY 5.6-like
*TraesCS5D02G498700*	-2.07	4.20E-15	-2.15	1.62E-11	protein NRT1/PTR FAMILY 5.6-like
*TraesCS6A02G267600*	-1.44	0.0001	-1.92	0.002	protein NRT1/PTR FAMILY 8.3-like
*TraesCS6A02G267700*	-3.00	1.90E-24	-1.56	6.45E-06	protein NRT1/PTR FAMILY 8.3-like
*TraesCS6A02G267800*	-3.23	3.26E-05	-2.90	0.006	protein NRT1/PTR FAMILY 8.3-like
*TraesCS6A02G268500*	-1.09	7.42E-05	-1.56	0.005	protein NRT1/PTR FAMILY 8.3-like
*TraesCS6B02G295000*	-2.41	1.52E-13	-2.40	5.56E-08	protein NRT1/PTR FAMILY 8.3-like
*TraesCS6B02G295600*	-1.85	1.83E-12	-1.78	0.005	protein NRT1/PTR FAMILY 8.3-like
*TraesCS6D02G247000*	-4.10	4.91E-25	-2.03	0.0001	protein NRT1/PTR FAMILY 8.3-like
*TraesCS6D02G247100*	-1.45	9.32E-05	-1.88	5.83E-05	protein NRT1/PTR FAMILY 8.3-like isoform X1
*TraesCS7D02G378300*	1.53	0.0004	1.04	0.008	protein NRT1/PTR FAMILY 8.3-like isoform X1

NN and LN indicate normal- and low-nitrogen conditions, respectively. AA and BB indicate the NIL^KN9204^ and the NIL^J411^, respectively. The expression levels between the NIL^KN9204^and the NIL^J411^ were compared under each condition, with the latter as a control. The expression ratios are presented as log_2_FC values. Genes with a log_2_FC ≤ -1 (p-adjusted< 0.05, coloured blue) represent the genes whose expression was downregulated in the NIL^KN9204^, and genes with log_2_FC ≥ 1 (p-adjusted< 0.05, coloured red) represent the genes whose expression was upregulated in the NIL^KN9204^.

Phytohormones, together with their receptors and signaling components, constitute the main intrinsic regulatory pathways of root traits. Ethylene, whose biosynthesis is regulated by several key enzymes, such as 1-aminocyclopropane-1-carboxylic acid (ACC) synthase (ACS) and ACC oxidase (ACO), usually plays a negative regulatory role in root growth, inhibiting root enlargement and root meristem cell proliferation (Pattyn et al., 2020). In addition, ethylene regulates the expression of glutathione S-transferase (GST)-encoding genes. GST catalyses the conjugation of glutathione to various electrophilic compounds involved in the oxidative stress response, crosstalk with other hormones and drought-induced root growth (Dalal et al., 2018). Under both NN and LN conditions, significant increases in expression levels of genes encoding ACOs and GSTs were detected in the NIL^J411^, with parallel increases in expression levels of genes encoding ethylene-responsive TFs (**Table 4**), implying that enhanced ethylene signaling together with glutathione metabolism negatively regulated root growth in the NIL^J411^. GA, whose catabolism is catalysed by GA2-oxidase, is considered a negative regulator of lateral root formation (Gou et al., 2010). However, overexpression of GA2-oxidase-encoding genes led to shortened primary root length, and mutations in these kinds of genes led to somewhat longer roots (Li et al., 2017), indicating a negative correlation between GA2-oxidase-encoding genes and root length. Compared to the NIL^KN9204^, the NIL^J411^ displayed upregulated expression levels of several GA2-oxidase-encoding genes under both NN and LN conditions (**Table 4**); these upregulated levels may have inhibitory effects on root development. Brassinosteroids (BRs) regulate both root meristem size and cell expansion in controlling root growth. Deficiency or insensitivity of BRs leads to reduced root growth and lateral root formation (Wei and Li, 2016). The decreased expression of receptor-like protein kinase BRI1-like 3 genes in the NIL^J411^ might impair BR signaling and inhibited root enlargement (**Table 4**). Auxin facilitates lateral root initiation and development (Fukaki and Tasaka, 2009), while cytokinin antagonistically negatively regulates root growth (Moubayidin et al., 2009). In rice, crown root primordium initiation was promoted by auxin signaling, whereas cytokinin signaling inhibited it (Neogy et al., 2021). Members of the *GH3* gene family respond to auxin and their encoding products use various amino acids as substrates to form indole-acetic acid (IAA)-amido conjugates for temporary storage and degradation (Staswick et al., 2005). Arabidopsis *GH3.17* gene regulates environment-induced hypocotyl elongation (Zheng et al., 2016) and it is also responsible for root elongation, whereas gh3 mutants accumulate more free-IAA at the expense of any IAA-Glu and IAA-Asp auxin conjugates which causes defects in root development, demonstrating the critical roles of *GH3* genes in auxin homeostasis and plant development (Guo et al., 2022). Cytokinin is activated by phosphoribohydrolase, which catalyses the hydrolysis of the bond between N*^6^*-substituted bases and ribose 5’-monophosphates in cytokinin precursors during its biosynthesis process (Kurakawa et al., 2007). Rice *GH3-8* gene functions as a repressor of auxin-dependent developmental signaling, overexpression of *GH3-8* suppressed auxin action, which resulted in abnormal morphology similar to that reported in auxin-deficient plants i.e. shorter roots and fewer adventitious roots (Ding et al., 2008). Significant increases in expression levels were observed for genes encoding GH3.8 and phosphoribohydrolase in the NIL^J411^, accompanied by decreased expression levels of a gene encoding an auxin-responsive protein (**Table 4**), implying the presence of weakened auxin signaling and accelerated cytokinin flux in the NIL^J411^, which may be related to the differences in root traits of the pairwise NILs.

**Table 4:** DEGs involved in phytohormone metabolism and signaling between the pairwise NILs.

Gene_ ID	log_2_FC (NN_NIL^KN9204^/NN_NIL^J411^)	*p*-adjusted	log_2_FC (LN_NIL^KN9204^/LN_NIL^J411^)	*p*-adjusted	NR_description
Ethylene					
*TraesCS1A02G370400*	-5.05	0.0004	-2.53	5.63E-05	ethylene-responsive transcription factor ERF109-like
*TraesCS1A02G370600*	-3.55	4.19E-17	-1.44	8.02E-05	AP2 domain containing protein
*TraesCS1A02G370700*	-5.28	1.08E-51	-2.08	8.05E-11	ethylene-responsive transcription factor ERF109-like
*TraesCS1B02G117400*	-1.32	5.13E-05	-1.44	6.19E-09	1-aminocyclopropane-1-carboxylate oxidase-like
*TraesCS1B02G389700*	-3.15	3.17E-20	-1.49	6.98E-06	ethylene responsive transcription factor 6
*TraesCS1B02G389800*	-3.64	1.61E-26	-1.78	1.94E-07	ethylene-responsive transcription factor ERF109-like
*TraesCS1D02G098000*	-1.80	2.67E-11	-1.77	5.13E-10	1-aminocyclopropane-1-carboxylate oxidase-like
*TraesCS1D02G098100*	-1.10	8.10E-06	-1.51	7.25E-09	1-aminocyclopropane-1-carboxylate oxidase-like
*TraesCS1D02G376500*	-4.11	8.13E-33	-1.79	7.68E-08	ethylene-responsive transcription factor ERF109-like
*TraesCS1D02G376600*	-3.26	2.22E-37	-1.33	0.0001	ethylene-responsive transcription factor ERF109-like
*TraesCS1D02G376700*	-3.34	1.30E-23	-1.36	2.22E-05	ethylene-responsive transcription factor ERF109-like
*TraesCS1D02G376800*	-4.79	1.53E-42	-2.08	0.0005	ethylene-responsive transcription factor ERF109-like
*TraesCS2D02G397100*	-5.47	0.0002	-2.92	4.59E-09	ethylene-responsive transcription factor ERF025-like
*TraesCS3B02G158800*	1.56	0.037	1.89	0.003	AP2 domain transcription factor TaDREB2
*TraesCS3B02G412500*	-2.39	4.76E-05	-1.17	0.046	F-box domain containing protein, expressed
*TraesCS4A02G256500*	-4.94	5.60E-08	-4.57	5.50E-19	1-aminocyclopropane-1-carboxylate synthase 1-like
*TraesCS4B02G058200*	-2.51	7.59E-09	-3.30	5.16E-23	1-aminocyclopropane-1-carboxylate synthase 1-like
*TraesCS4D02G058200*	-2.67	1.16E-12	-2.89	1.08E-23	1-aminocyclopropane-1-carboxylate synthase 1-like
*TraesCS4D02G096400*	-1.62	8.07E-10	-1.25	2.91E-11	lipase-like PAD4
*TraesCS5B02G236900*	-2.68	6.45E-17	-1.25	0.0009	ethylene-responsive transcription factor ERF109-like
*TraesCS5D02G245300*	-2.37	2.30E-10	-1.34	0.002	ethylene-responsive transcription factor ERF109-like
*TraesCS6A02G235100*	1.95	5.71E-07	1.08	0.004	predicted protein
*TraesCS6A02G256600*	-3.44	9.28E-14	-2.30	4.23E-12	ethylene-responsive transcription factor ERF027-like
*TraesCS6A02G319500*	-3.88	2.62E-11	-2.36	1.01E-09	ethylene-responsive transcription factor ERF109-like
*TraesCS6A02G330500*	-1.83	5.11E-16	-1.26	1.25E-06	ethylene-responsive transcription factor ERF014-like
*TraesCS6B02G268700*	-3.01	3.89E-17	-2.22	1.77E-19	ethylene-responsive transcription factor ERF027-like
*TraesCS6B02G281000*	-1.76	4.23E-09	-1.36	1.78E-07	ethylene-responsive transcription factor 1-like
*TraesCS6B02G350100*	-4.04	9.00E-12	-2.71	5.23E-10	ethylene-responsive transcription factor ERF109-like
*TraesCS6B02G350400*	-3.41	7.85E-42	-1.71	5.20E-07	ethylene-responsive transcription factor ERF109-like
*TraesCS6B02G361400*	-2.21	9.84E-22	-1.05	9.84E-08	ethylene-responsive transcription factor ERF014-like
*TraesCS6D02G217800*	1.72	1.14E-05	1.02	0.0004	ethylene-responsive transcription factor ERF054-like
*TraesCS6D02G237800*	-3.23	2.79E-19	-2.32	1.03E-21	ethylene-responsive transcription factor ERF027-like
*TraesCS6D02G298700*	-3.68	1.54E-06	-2.41	3.40E-09	ethylene-responsive transcription factor ERF109-like
*TraesCS6D02G299000*	-4.6422	0.007	-2.09	0.0022	ethylene-responsive transcription factor ERF109-like
*TraesCS6D02G299100*	-3.71	2.77E-37	-1.96	2.11E-06	ethylene-responsive transcription factor ERF109-like
*TraesCS6D02G309600*	-1.66	1.08E-15	-1.13	3.56E-05	ethylene-responsive transcription factor ERF014-like
Glutathione S-transferase					
*TraesCS1B02G194100*	-1.22	0.002	-1.11	0.0006	putative glutathione S-transferase GSTU6
*TraesCS1B02G194500*	-1.36	0.0005	-1.25	0.046	probable glutathione S-transferase GSTU6
*TraesCS2A02G218700*	-4.72	9.96E-19	-2.78	5.22E-20	probable glutathione S-transferase GSTU6
*TraesCS2B02G244100*	-3.87	3.27E-19	-2.39	6.06E-19	probable glutathione S-transferase GSTU6
*TraesCS2B02G322800*	-1.38	1.14E-06	-1.19	9.82E-05	glutathione S-transferase TCHQD
*TraesCS2D02G044100*	-3.99	0.03	-2.58	0.002	probable glutathione S-transferase GSTF1
*TraesCS2D02G224700*	-1.83	0.001	-1.72	5.55E-08	probable glutathione S-transferase GSTU6
*TraesCS2D02G304500*	-1.58	9.35E-06	-1.45	8.40E-12	glutathione S-transferase TCHQD
*TraesCS3A02G309100*	-1.92	5.18E-06	-1.14	4.15E-05	unnamed protein product
*TraesCS3D02G130700*	1.39	0.006	1.16	0.0006	probable glutathione S-transferase GSTU6
*TraesCS3D02G486100*	-1.15	9.69E-05	-1.07	3.18E-07	glutathione transferase GST 23-like
Gibberellin					
*TraesCS1A02G334400*	-1.81	2.73E-09	-2.32	3.52E-16	gibberellin 2-oxidase
*TraesCS2A02G189600*	-1.61	3.52E-11	-1.29	5.11E-09	chitin-inducible gibberellin-responsive protein 2-like
*TraesCS2B02G217500*	-1.59	8.03E-14	-1.32	1.55E-09	chitin-inducible gibberellin-responsive protein 2-like
*TraesCS2B02G239400*	-1.24	5.27E-08	-1.03	3.57E-05	chitin-inducible gibberellin-responsive protein 1
*TraesCS2D02G198200*	-1.36	5.50E-11	-1.46	1.23E-10	chitin-inducible gibberellin-responsive protein 2-like
*TraesCS2D02G220200*	-1.33	2.10E-10	-1.03	9.21E-07	chitin-inducible gibberellin-responsive protein 1
*TraesCS3A02G122600*	1.25	0.04	1.67	3.54E-07	gibberellin 3-oxidase 2-1
*TraesCS3A02G294000*	-1.73	2.71E-09	-1.45	9.93E-17	gibberellin 2-oxidase
*TraesCS3B02G328700*	-1.86	1.62E-08	-1.71	3.62E-07	gibberellin 2-oxidase 3 isozyme B1
*TraesCS6A02G221900*	-2.35	1.36E-13	-2.00	1.46E-10	gibberellin-2-oxidase-A9
*TraesCS6D02G213100*	-2.84	2.58E-08	-3.35	3.80E-17	gibberellin 2-beta-dioxygenase 8-like
Brassinosteroid					
*TraesCS2A02G142300*	1.42	4.85E-05	1.077	7.91E-05	probable cytochrome P450 313a4
*TraesCS3B02G550900*	-2.31	0.004	-1.70	0.0004	protein BRASSINOSTEROID INSENSITIVE 1-like isoform X1
*TraesCS5B02G174400*	1.24	9.18E-07	1.43	1.95E-07	receptor-like protein kinase BRI1-like 3
*TraesCS5D02G181500*	1.32	1.42E-07	1.56	4.46E-14	receptor-like protein kinase BRI1-like 3
Auxin					
*TraesCS2A02G183900*	-1.73	1.42E-12	-1.43	1.77E-13	probable indole-3-acetic acid-amido synthetase GH3.8
*TraesCS5A02G378300*	1.42	0.0498	1.12	3.10E-05	Auxin-responsive protein IAA30
*TraesCS6B02G359400*	-1.38	0.001	-1.07	0.005	predicted protein, partial
Cytokinin					
*TraesCS1A02G156100*	-3.19	2.83E-07	-2.22	3.79E-14	probable cytokinin riboside 5’-monophosphate phosphoribohydrolase LOGL10 isoform X1
*TraesCS3D02G475800*	-2.54	4.52E-07	-1.43	6.95E-05	cytokinin dehydrogenase 4-like

NN and LN indicate normal- and low-nitrogen conditions, respectively. AA and BB indicate the NIL^KN9204^ and the NIL^J411^, respectively. The expression levels between the NIL^KN9204^ and the NIL^J411^ were compared under each condition, with the latter as a control. The expression ratios are presented as log_2_FC values. Genes with a log_2_FC ≤ -1 (p-adjusted< 0.05, coloured blue) represent the genes whose expression was downregulated in the NIL^KN9204^, and genes with log_2_FC ≥ 1 (p-adjusted< 0.05, coloured red) represent the genes whose expression was upregulated in the NIL^KN9204^.

Several types of TFs play essential roles in root growth by regulating the expression of downstream genes that are usually closely associated with nutrient acquisition. Overexpression of *TaNFYA-B1* was shown to increase both primary root length and total lateral root length in wheat (Qu et al., 2015). The expression levels of genes encoding NF-Y subunit C-3-like proteins (*TraesCS1A02G354900*, *TraesCS1B02G366800* and *TraesCS1D02G355600*) were higher in the NIL^KN9204^ than in the NIL^J411^ under LN condition (**Table 5**), indicating their potential contribution to LN-induced root enlargement. NAC TFs function in response to $NO_{3}^{-}$ , depending on the NRT1.1 $NO_{3}^{-}$ transport function (Vidal et al., 2014), to increase root biomass (He et al., 2015). Two genes encoding NAC TFs (*TraesCS3A02G078400* and *TraesCS3D02G078900*) exhibited higher expression levels in the NIL^KN9204^ than in the NIL^J411^ under both NN and LN conditions (**Table 5**), implying that they had similar effects on root growth. Ethylene responsive factors (ERFs) belong to the AP2/ERF (APETALA2/ERF) superfamily of transcription factors associated with various developmental processes and stress tolerance. It was reported that ERF transcription factor gene *TaSRL1* resulted in short root length when ectopically expressed in rice (Zhuang et al., 2021). In the present study, expressions of ERF-like genes were significantly down-regulated in the NIL^KN9204^ compared to the NIL^J411^ under both NN and LN conditions, which may contribute to longer root length in NIL^KN9204^.

**Table 5:** Differentially expressed TF-encoding genes between the pairwise NILs.

Gene_ ID	log_2_FC (NN_NIL^KN9204^/NN_NIL^J411^)	*p*-adjusted	log_2_FC (LN_NIL^KN9204^/LN_NIL^J411^)	*p*-adjusted	NR_description
NTF					
*TraesCS1A02G354900*	0.70	1	2.16	0.02	nuclear transcription factor Y subunit C-3-like
*TraesCS1B02G366800*	0.64	0.89	1.83	0.0007	nuclear transcription factor Y subunit C-3-like
*TraesCS1D02G355600*	0.45	0.95	2.60	0.003	nuclear transcription factor Y subunit C-3-like
NAC					
*TraesCS3A02G078400*	1.48	0.004	1.37	3.04E-07	NAC transcription factor
*TraesCS3A02G339600*	-3.07	3.15E-22	-3.01	1.27E-27	NAC transcription factor 4
*TraesCS3D02G078900*	1.79	6.26E-06	1.39	5.36E-05	NAC transcription factor

NN and LN indicate normal- and low-nitrogen conditions, respectively. AA and BB indicate the NIL^KN9204^ and the NIL^J411^, respectively. The expression levels between the NIL^KN9204^ and the NIL^J411^ were compared under each condition, with the latter as a control. The expression ratios are presented as log_2_FC values. Genes with a log_2_FC ≤ -1 (p-adjusted< 0.05, coloured blue) represent the genes whose expression was downregulated in the NIL^KN9204^, and genes with log_2_FC ≥ 1 (p-adjusted< 0.05, coloured red) represent the genes whose expression was upregulated in the NIL^KN9204^.

In conclusion, transcriptomic analysis in the present study revealed the landscape of the DEGs between the *QMrl-7B* NILs under both NN and LN conditions, reflecting the physiological basis of the large roots of the superior parent KN9204. This study used pairwise NILs for analysis to dampen the effects from different genetic backgrounds; as such, the DEGs are more likely to be related to *QMrl-7B*, either directly or indirectly. These results revealed many pathways regulated by genes that might interact genetically with *QMrl-7B*, which may provide a foundation for the application of *QMrl-7B* in molecular breeding. However, since the materials used for RNA-seq were seedlings and not mature plants, much work is still needed to elucidate the overall genetic network that controls ideal root traits of wheat plants at different stages.

## Data availability statement

The data presented in the study are deposited in the NCBI repository, accession number PRJNA889686. The accession number PRJNA889686 (http://www.ncbi.nlm.nih.gov/bioproject/889686).

## Author contributions

LS, JJL, LZ, NZ, WZ, DM, XR, JJ and JML conducted the hydroponic culture and field experiments. LS, JJL, LZ and AB analyzed the data. LS, JJL, YN and AB wrote the manuscript. RL and JML revised the manuscript. All authors contributed to the article and approved the submitted version.

## Funding

This research was jointly supported by grants from the National Key Research and Development Program of China (2021YFF1000403), the National Natural Science Foundation of China (U22A6009), Hebei Natural Science Foundation (C2021205013) and China Agriculture Research System of MOF and MARA (CARS-03).

## Acknowledgments

We are very grateful to Dr. Xigang Liu from Hebei Normal University, Shijiazhuang, China for his critical review of the manuscript.

## Conflict of interest

The authors declare that the research was conducted in the absence of any commercial or financial relationships that could be construed as a potential conflict of interest.

## Publisher’s note

All claims expressed in this article are solely those of the authors and do not necessarily represent those of their affiliated organizations, or those of the publisher, the editors and the reviewers. Any product that may be evaluated in this article, or claim that may be made by its manufacturer, is not guaranteed or endorsed by the publisher.

## Abbreviations

NN, normal-nitrogen; LN, low-nitrogen; NIL, near-isogenic line; DEG, differentially expressed gene; QTL, quantitative trait locus; RNA-seq, RNA sequencing; NUE, nitrogen use efficiency; MRL, maximum root length; TRL, total root length; RA, root surface area; RV, root volume; GO, Gene Ontology; KEGG, Kyoto Encyclopedia of Genes and Genomes.
