# Supplementary figures and images for: Transcriptomic analysis reveals the contribution of QMrl-7B to wheat root growth and development
Source: Front Plant Sci. 2022 Nov 14;13:1062575. doi: 10.3389/fpls.2022.1062575 (PMC9706392; doi:10.3389/fpls.2022.1062575)

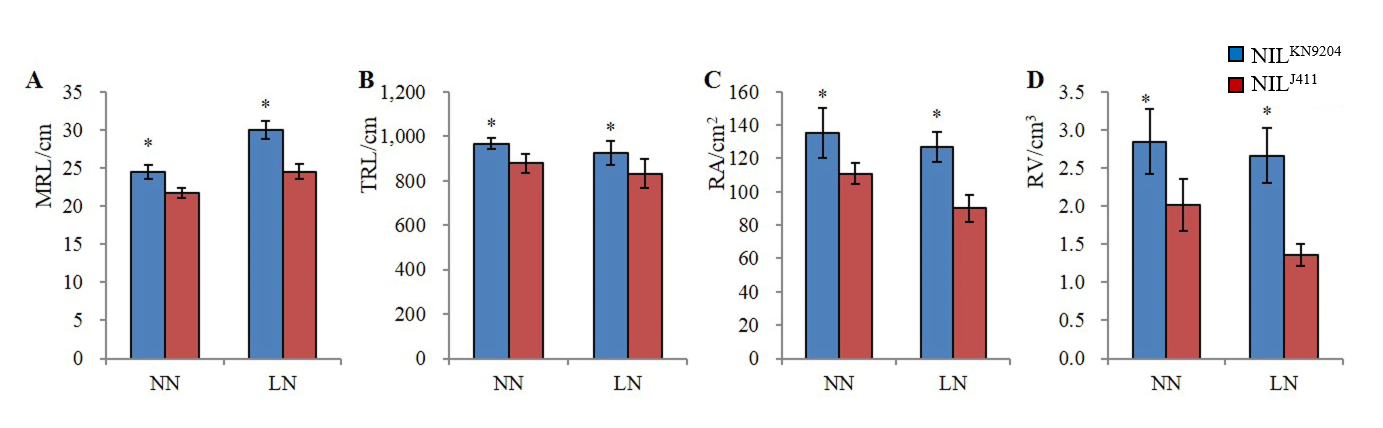

Supplement: Supplementary file 1 [file Image_1.tif]

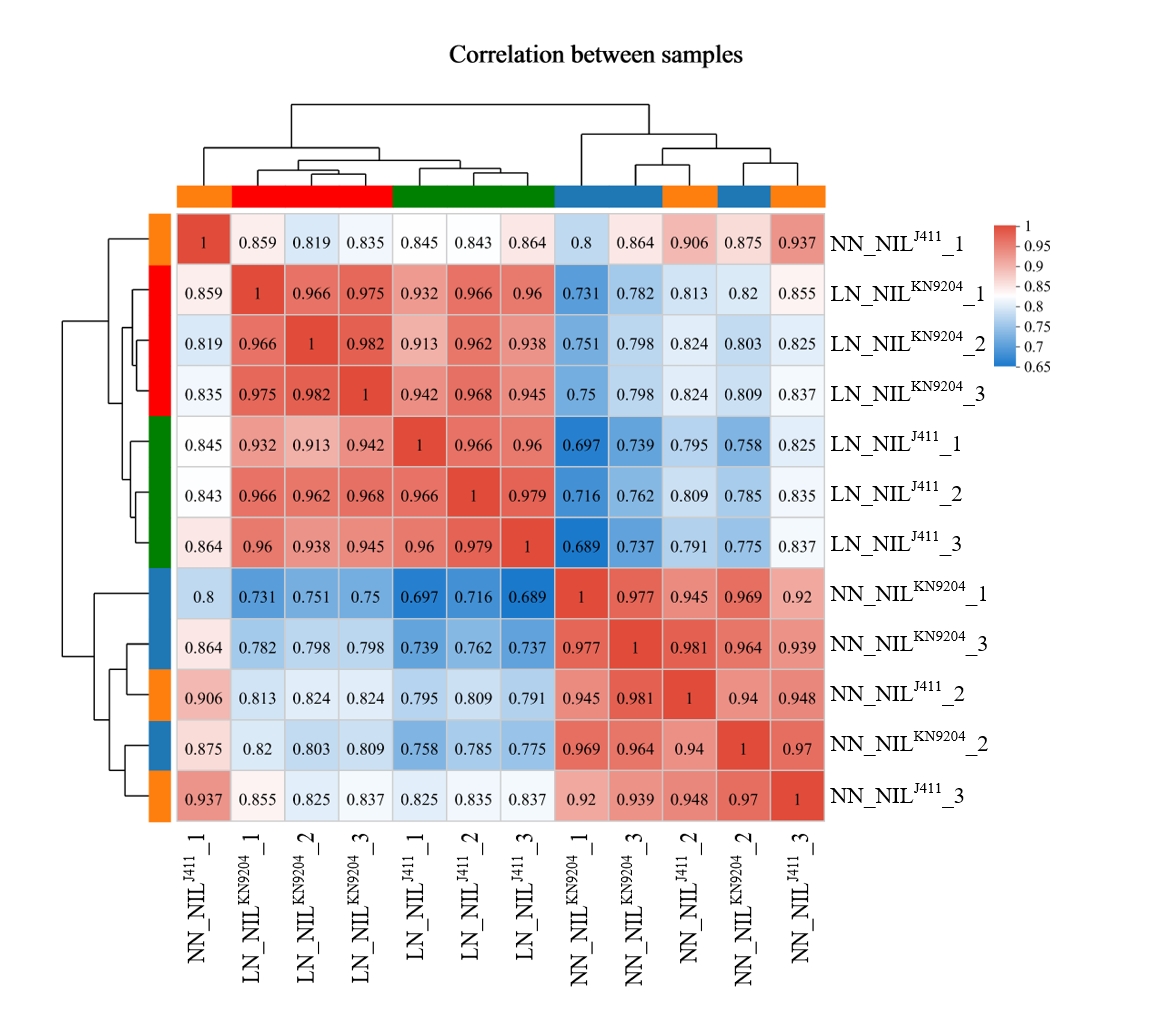

Supplement: Supplementary file 2 [file Image_2.tif]
